# Urban public services and fertility intentions of internal migrants in China

**DOI:** 10.1371/journal.pone.0300345

**Published:** 2024-03-28

**Authors:** Sichen Liu, Quanling Cai, Mingxing Wang, Kaisheng Di

**Affiliations:** 1 College of Economics and Management, Wuhan University, Wuhan, Hubei Province, China; 2 Department of Management and Economics, Tianjin University, Tianjin, China; 3 College of Politics and Public Administration, Qinghai Minzu University, Xining, Qinghai Province, China; 4 College of Finance and Economics, Qinghai University, Xining, Qinghai Province, China; 5 Department of Party Committee, Party School of the Qinghai Provincial Committee of CPC, Xining, Qinghai Province, China; Hosei University: Hosei Daigaku, JAPAN

## Abstract

As China continues to implement its progressive fertility promotion policy, there has been a drastic decline in the fertility rate. Given that the migrant population constitutes more than a quarter of China’s total population, enhancing the willingness of this demographic to have additional children through policy-guided urban public services is pivotal for optimizing China’s population development strategy. This study analyzes the influence of urban public services on the reproductive intentions of the migrant population, utilizing data from 110,667 migrant families with one child, drawn from China’s Migrant Population Dynamic Monitoring data in 2016 and 2018. The data analysis reveals several key findings: (1) Urban public services, overall, exhibit a notable positive effect on the willingness of the migrant population to have more children, albeit with limitations and a declining trend. (2) Among urban public services, primary basic education significantly impacts the willingness of the migrant population to expand their families. (3) Large cities have created a ’reverse screening’ effect on the migrant population, leading to differential access to public services. This scenario caters effectively to the high human capital migrant individuals while reducing accessibility to livelihood public services for the low human capital migrant population. This paper critically evaluates China’s progressively adjusted fertility policy from the perspective of the migrant population. It underscores the necessity of establishing a comprehensive fertility support policy system across China.

## Introduction

In recent years, experts and scholars have increasingly recognized socio-economic development as a crucial factor in reducing global fertility levels [1]. As Polanyi noted, a spontaneously regulated market has never existed. Since the industrial era [2], developed countries have witnessed concurrent advancements in medical technology [3], increased educational costs [4], and a substantial expansion of social security coverage [5]. Changes in societal attitudes and approaches [6], along with decreasing infant and child mortality rates and the escalating costs of child-rearing [7], have collectively influenced a shift in attitudes towards childbearing, resulting in a decline in birth rates [8]. This economic and social "two-way street," signifying the expansion of market forces [9], ultimately triggers a counter-movement to safeguard human welfare [10]. According to United Nations statistics, the average urbanization level in developed countries is projected to reach 81.3 percent in 2020, accompanied by an ultra-low average total fertility rate (below 1.3). Contrarily, as per the results of the 7th China Population Census, China’s total fertility rate for women of childbearing age is expected to be 1.3 in 2020, already at an ultra-low level, despite the degree of urbanization lagging significantly behind that of developed countries [11, 12].

Since the initiation of reform and opening up, China has undergone swift urbanization [13], witnessing a rise in the urbanization rate from 17.9% in 1978 to 64.31% in 2021. The National Development and Reform Commission (NDRC) of China highlighted that, with the gradual stabilization of China’s Migrant Population, each one percentage point increase in this demographic implies a migration of 14 million people from rural areas to urban centers [14]. Consequently, China’s year-end resident population has surged from 1,143.33 million people in 1990 to 1,412.6 million people by the end of 2021 [15]. The results of the 7th National Population Census reveal that by the conclusion of 2021, China’s Migrant Population has reached 385 million, constituting more than a quarter of the country’s total population. As per the Statistical Bulletin of the National Economic and Social Development of the People’s Republic of China [16], as of the end of 2021, the nation had 504 million people separated from their households, of which 385 million were mobile, making up more than a quarter of the total population [5]. The 2020 Seventh Population Census data indicates that women of childbearing age accounted for 22.86% of China’s total population [17], with the proportion of women of childbearing age among the population separated from households reaching 16.80%. Concurrently, the proportion of women of childbearing age within the Migrant Population amounted to 12.56% of the total population, totaling 177 million [18]. Leveraging this substantial base of women of childbearing age in the Migrant Population, China conducted a nationwide survey on the dynamic monitoring of health and family planning within this group [19]. The survey revealed that following the implementation of the "comprehensive two-child" policy, barring a minor, transient surge in the birth rate in 2016 attributed to compensatory childbearing, the overall fertility intention in 2018 declined by 10% compared to 2016. Increasingly, families within the Migrant Population, adopting a cautious stance toward the fertility policy, opted to forgo their intention of "having the next child [20]".

In the context outlined above, this paper aims to address three pivotal questions. Firstly, it seeks to assess the current state of urban public services accessible to the Migrant Population, considering the recent improvements in these services. Secondly, the research aims to identify the urban public service indicators that significantly influence the Migrant Population’s willingness to have another child. Thirdly, it aims to pinpoint the segment of the Migrant Population most responsive to urban public services and delve into its underlying rationale. To address these inquiries, the study leverages data from the 2016 and 2018 national mobile population dynamics monitoring (referred to as CMDS2016 and CMDS2018) conducted by the National Health Commission. Additionally, it utilizes data from the 2016 and 2018 China Urban Statistical Yearbooks and data from the statistical yearbooks of various provinces and municipalities on the resident population during the same years. The objective is to comprehensively examine the urban public services accessible to the mobile population at the place of inflow. Building on this analysis, the study aims to profoundly investigate the impact and mechanisms of urban public services on the Migrant Population’s inclination to have another child. Furthermore, the research intends to contribute insights into the development of a birth-friendly urban public service system for the Migrant Population, offering valuable guidance for advancing the establishment of a comprehensive public service system in China.

## Literature review and theoretical foundations

### Literature review

According to the "sequential pattern" theory of reproductive intention [21], the reproductive intentions of China’s Migrant Population exhibit dynamic changes [22]. The traditional fertility notions prevalent in the place of origin [23], such as "more children, more happiness" [24] and "passing on the family line" [25], have historically influenced the Migrant Population. Simultaneously, they are confronted with modern fertility concepts like "female independence" [26] and "fewer children, better births" [27] in the destination area. Consequently, some scholars posit that the willingness of the Migrant Population to have children is lower than that in the place of origin but higher than that in the destination area [28].

While earlier studies [12] in China indicated that the reproductive intentions of the Migrant Population were primarily shaped by fertility policies [29], recent adjustments in China’s fertility policy suggest a convergence between the reproductive intentions of the Migrant Population and the genuine intentions of their families [13]. Previous literature predominantly focused on analyzing the impact on the fertility intentions of the Migrant Population from the perspectives of individual and family characteristics [30]. Regarding individual characteristics, an increase in educational attainment weakens traditional childbearing beliefs, significantly decreasing childbearing intentions among highly educated female migrants [31]. Male migrants, with improved education levels experience delayed childbearing intentions [32], forming a "U" shaped curve [33]. The influence of economic factors, such as income levels, on fertility intentions and behaviors is perceived as complex, with some studies suggesting that a short-term increase in income levels can promote fertility intentions [34].

In terms of family characteristics, analysis of "single," "doubly independent," and "doubly non-permanent" family groups based on 2014 Migrant Population data reveals significantly higher fertility rates compared to those in the 1980s [12]. Conversely, a more recent analysis of these family groups based on 2014 Migrant Population data indicates uniformly low fertility rates [35]. Scholars posit that the Migrant Population, influenced by external factors such as traditional rural concepts and elder influence, tends to make reproductive decisions navigating between the contrasting fertility cultures of urban and rural settings [36]. Moreover, some scholars argue that the regional culture at the destination and the socio-economic status of the fam region’s economic development level differentially impact the Migrant Population’s willingness population to have more children [37].

Aligned with the global trend of establishing a service-oriented government, the emphasis on public service theory has gained prominence among a growing cohort of experts and scholars [38]. Scholars [39], in their endeavor to dissect the fertility intentions of the Migrant Population, delve into the lens of urban integration [40]. They posit that Migrant Populations exhibiting stronger urban adaptation tend to manifest weaker fertility intentions [41]. The degree of integration with the destination area [42], juxtaposed with the native ties to the departure region [43], is perceived as exerting a direct influence on the fertility intentions of the Migrant Population [16]. Some scholars [44] take a novel approach by scrutinizing fertility policy support for both "birth" and "parenting" [45], advocating increased governmental support for the latter [46].

Against the backdrop of China’s recent adjustments to its progressive fertility policy [47], evident in Table 1, the creation of fertility-friendly cities has become a focal point on the government’s agenda [48]. Navigating from the viewpoint of policy support at the destination [49], the challenge lies in minimizing the costs associated with childbearing and augmenting the inclination to give birth [50]. This paradigm shift [51] underscores the pivotal role of the fertility support policy system [52]. According to China’s National New Urbanization Plan [53], the essential urban public services that the Migrant Population should access include basic education for accompanying children [54], employment and entrepreneurship services [55], basic social security [56], basic medical care [57], and guaranteed housing [58]. To understand the impact on the fertility intentions of the Migrant Population, this paper categorizes these services into two overarching types: safeguard services (encompassing social security like pension and medical insurance) and development services (encompassing public medical resources, basic public education, and public infrastructure).

**Table 1:** A compendium of the Chinese government’s progressive fertility policy adjustments over the past decade.

Period of time	Event	Policy	Main content
November 2013	Having a second child on one’s own	Decision of the Central Committee of the Communist Party of China on Several Major Issues Concerning Comprehensively Deepening Reforms	Adhering to the basic national policy of family planning, it has initiated the implementation of the policy that couples in which one of the parents is an only child may have two children.
January 2016	Comprehensive second-child policy	Law of the People’s Republic of China on Population and Family Planning	Article 18: "The State promotes the birth of two children per couple."
May 2021	Comprehensive three-child policy	Law of the People’s Republic of China on Population and Family Planning	Article 18 "The State promotes marriage and parenthood at the right age, and optimal parenthood. A couple may have three children."
June 2021	Top-level design of maternity support policies	Decision of the Central Committee of the Communist Party of China and the State Council on Optimizing Fertility Policy and Promoting Long-term Balanced Development of Population	Accelerating the construction of an active birth support policy system, and deepening and refining the overall plan and policy arrangements for more operational and active birth support measures.
August 2022	Constructing a fertility support policy system for the new stage of development	China’s National Health Commission and 17 other departments jointly issued the Guiding Opinions on Further Improving and Implementing Positive Birth Support Measures	Marriage, childbirth, upbringing and education are explicitly considered as a whole, and the supporting support measures for the three-child policy have been refined.

Note: The table is collected and organized by the author.

### Theoretical foundations

Two fundamental questions precede the inquiry into whether urban public services can enhance the fertility intentions of the Migrant Population: firstly, the accessibility of these services for migrants [59]; secondly, whether the utility derived from urban public services can amplify the fertility benefits for migrants [53].

The costs associated with childbearing for the Migrant Population encompass various aspects [60]. Primarily, there are costs unrelated to urban public services, such as the opportunity costs incurred by mothers compelled to relinquish employment or career advancements to care for their children [61]. Additionally, there are direct costs encompassing clothing, food, housing, and transportation incurred due to the presence of children [62]. Concurrently, fertility benefits are augmented by the "positive externalities" arising from urban public services.

Strictly speaking, urban public services function as quasi-public goods with a certain degree of exclusivity [63]. Consequently, the provision of these services doesn’t automatically translate into accessibility for the Migrant Population. In essence, the supply of public services is a necessary but not a sufficient condition for migrants to access them. The National New Urbanization Plan (2021–2035) and the Key Tasks of New Urbanization and Urban-Rural Integration Development in 2022 categorize urban public services theoretically into two types: livelihood-guaranteeing public services (e.g., children’s education and healthcare) and developmental public services (e.g., transportation, environment, and culture resulting from infrastructure development).

Historical and institutional factors have led to a dualized development path between urban and rural areas, causing an influx of excessive and high-quality education and healthcare resources into large cities [64]. Better education and healthcare services are pivotal for promoting the fertility of the Migrant Population [65]. However, the household registration system, linked to high housing prices in major cities, hinders access to urban public services for the majority of migrants [66]. China’s household registration system, governing resource allocation across critical domains like education, healthcare, and employment, significantly favors major urban centers like Beijing and Shanghai over other regions. This system intricately links privileges such as housing, consumption patterns (e.g., vehicle ownership), education, and social security, providing differential benefits contingent upon registered residency. At birth, individuals in China are mandated to inherit one parent’s household registration, with potential migration opportunities related to education and employment. Nevertheless, some local governments enforce migration restrictions and levy substantial fees for urban residency expansion.

This system obstructs access to urban public services through multifaceted mechanisms. Primarily, educational inequalities persist, affording urban registrants superior access to quality education, while rural or non-urban residents encounter limited resources and restricted enrollment prospects. Secondarily, employment prospects remain constrained for non-urban registrants due to prevailing discrimination or limited access to urban job markets. Tertiary, disparities persist in healthcare and social security services, favoring urban registrants with more comprehensive support compared to their non-urban counterparts. Migration restrictions and associated high costs exacerbate these disparities. Despite the possibility of relocating household registration, migration quotas and substantial fees for urban residency expansion curtail mobility and hinder access to urban public services. China’s household registration system perpetuates unequal access to urban public services, posing a significant impediment to urban development. The disparities in resources, education, employment, and healthcare between urban and non-urban registrants underscore the systemic inequalities arising from this system, impeding equitable access to critical urban amenities.

The implementation of the government’s household categorization policy has led to structural differentiation in public service provision between large, medium, and small cities. Recent reports on the Migrant Population and census data reveal continued clustering in mega and central cities, where the accessibility of livelihood-focused public services is severely hampered by the household registration system and settling points. Conversely, medium-sized and small cities exhibit an inefficient supply of such services despite robust provision and high accessibility [67].

Within this vast Migrant Population, which constitutes nearly a quarter of China’s populace [53], the ability to access public services in large cities becomes a critical consideration. The "talent introduction policy" in major cities, designed to attract high human capital, initiates a "reverse screening" process for migrants, elevating the threshold for low-cost migrants to access urban public services [59].

**Fig 1:**
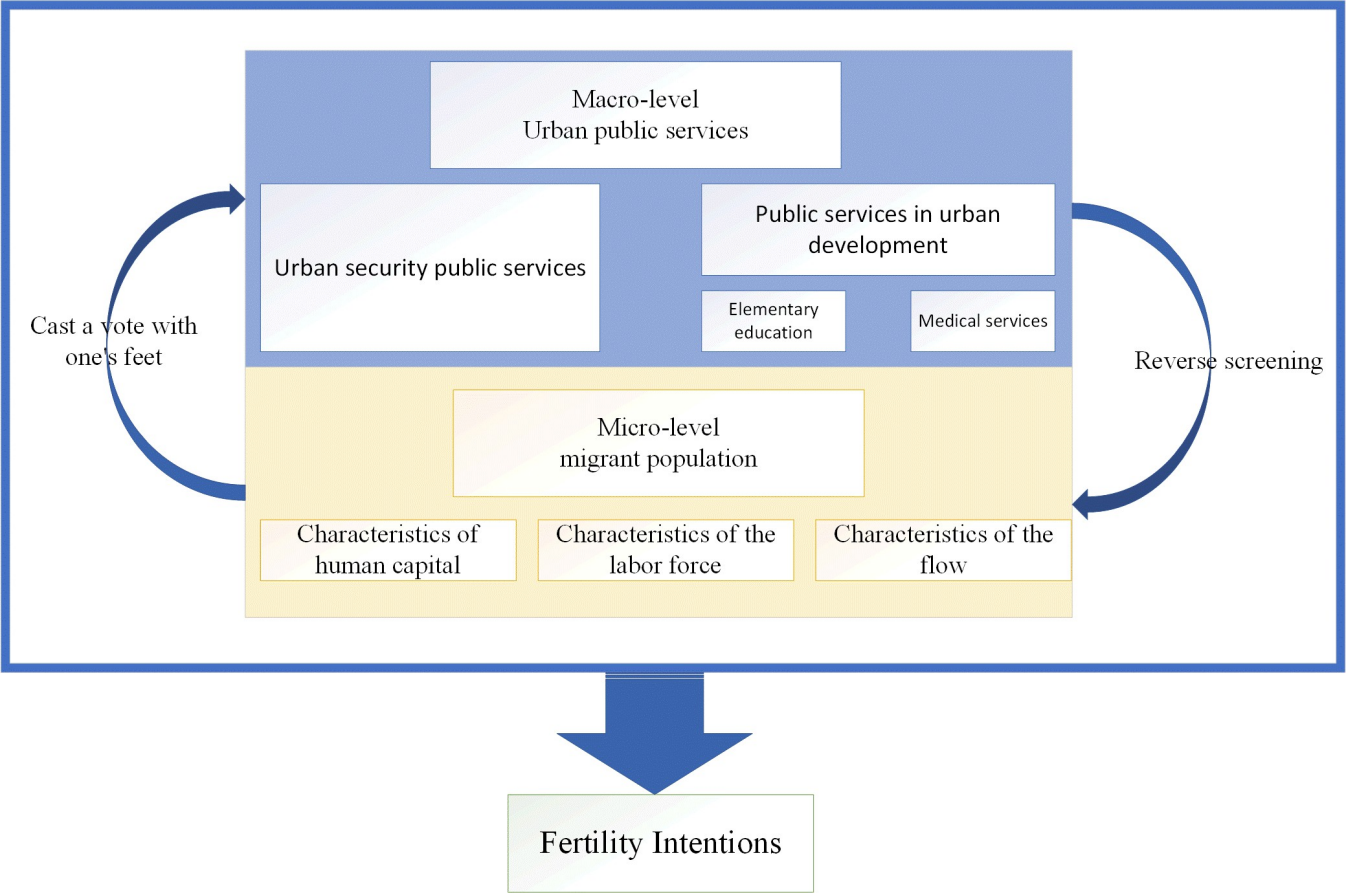
Research framework diagram.

**Fig 2:**
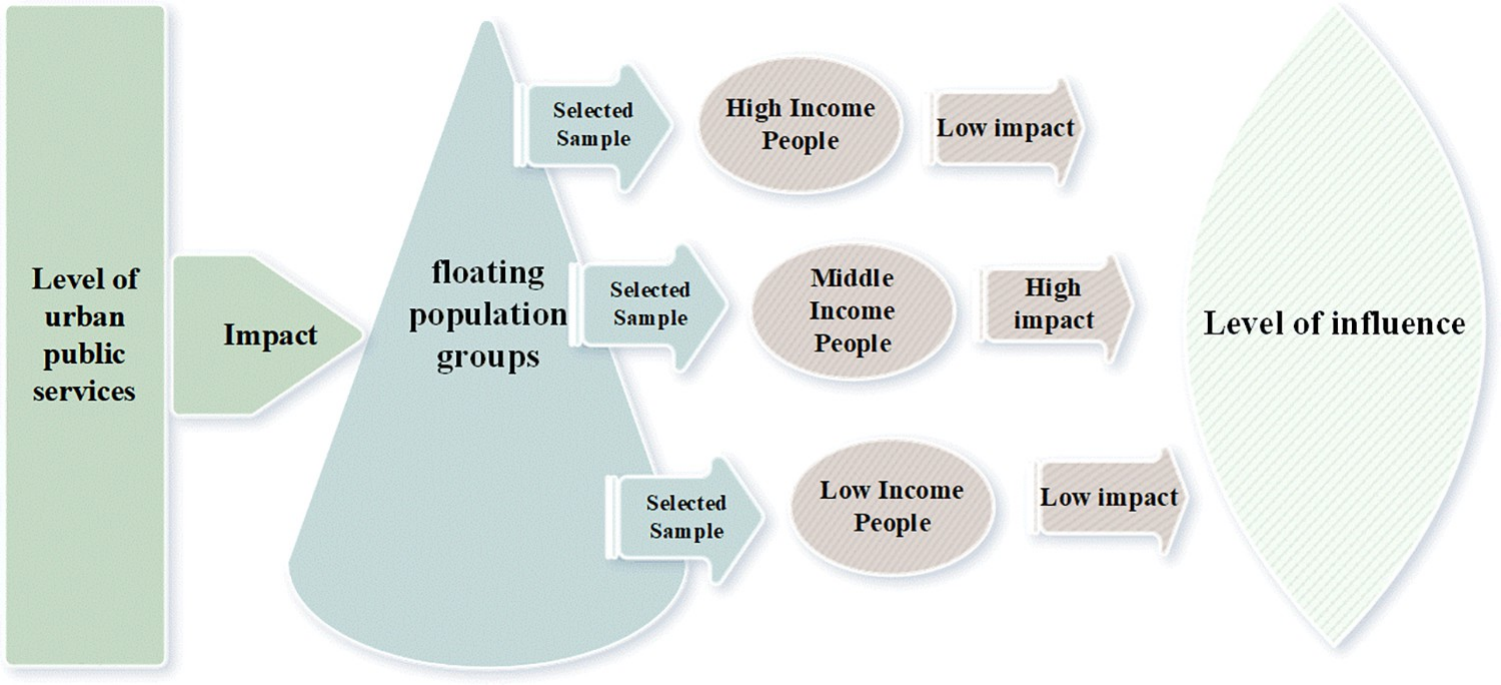
Heterogeneous effects of urban public services on the reproductive intentions of the migrant population. Note: Given the pyramidal structure of individual strata within the Migrant Population group, such as economic income and education level, the influence of received urban public services exhibits differentiation, manifesting a spindle-shaped structural characteristic.

Built upon this foundation, the study posits the following hypotheses(As shown in Figs 1 and 2):

H1: The comprehensive provision of urban public services can positively influence the willingness of the Migrant Population to have additional children.
H2: Smaller and medium-sized cities exert a more pronounced pull on the fertility intentions of the Migrant Population compared to larger cities.
H3: Public services falling within the livelihood protection category exert a more discernible impact on the fertility intentions of the Migrant Population than those categorized as infrastructure-related public services.
H4: Urban public service provision has a more significant impact on high-cost migrant populations compared to their low-cost counterparts. High-cost migrants, with their higher incomes and financial resources, are often able to afford and access better quality housing, education, healthcare, and other public services.

## Data and variable selection

### Data sources

In this study, we undertake several approaches. First, we employ CMDS data from 2016 and 2018 to assess the current status of the Migrant Population’s inclination toward having another child, focusing on a micro-individual perspective. Second, we utilize urban public services data from the China City Statistical Yearbook to gauge the level of public services across diverse cities from a macro perspective. Third, recognizing the substantial representation of the Migrant Population in the resident demographic of inflow areas, we collected data on the resident population at the end of 2015 and 2017. This data is sourced from the 2015, 1% Population Sample Survey Data of Chinese Provinces and Regions, statistical yearbooks of Chinese provinces and cities, and statistical bulletins on national economic and social development for each province and city. Fourth, to mitigate endogeneity concerns arising from survey timing, we introduce a lag of one year for the data on urban characteristics and resident population data, given that the survey for China’s mobile population health and family planning dynamic monitoring data occurs in the first half of the survey year. Fifth, considering data timeliness and the impact of the COVID-19 pandemic, we select survey data from 2016 and 2018 as our samples to investigate the willingness of the Migrant Population to reproduce. The survey spans 31 provinces (municipalities and autonomous regions), 106 cities (comprising provincial capitals, planned cities, prefecture-level cities, and county-level cities), 2,456 streets, and 4,912 neighborhood committees or administrative villages nationwide. A total of 170,000 samples were collected, covering respondents’ basic information, family details, work particulars, social security status, and intentions regarding residency and having another child.

### Sample structure

The following criteria were applied for data selection: 1. Exclusion of the Migrant Population with Hukou statuses (The household registration status of individuals or families in a specific area, which includes population statistics, migration records, and eligibility for access to public services and entitlements.) categorized as agriculture-to-resident, non-agriculture-to-resident, resident, and other samples, retaining only those with agricultural and non-agricultural Hukou to facilitate differentiation between rural-urban and urban-urban Migrant Populations. 2. Removal of samples whose reason for mobility is military enlistment to ensure comparability across sample types. 3. Elimination of samples involving out-of-country mobility, focusing exclusively on inter-provincial and intra-provincial mobility cases. 4. Concentration on the study of the Migrant Population with a spouse who has one child and is of childbearing age, equipped to express the desire to have another child.

**Fig 3:**
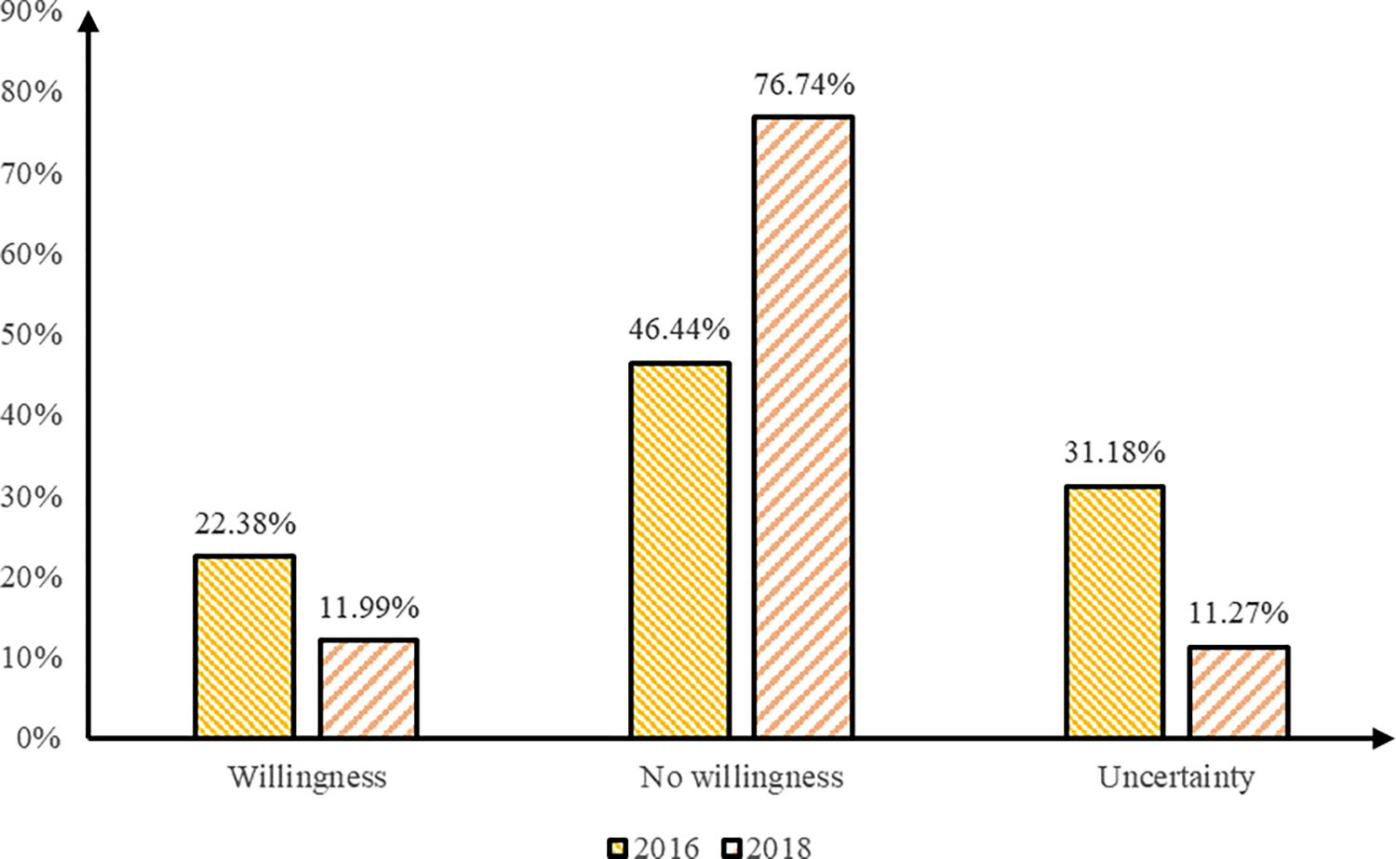
Comparing the overall willingness to have more children among the migrant population in 2016 and 2018. Notes: Data were from China’s Migration Dynamic Surveillance (2016) and China’s Migration Dynamic Surveillance (2018). This figure indicates that the willingness of the migrant population to have a second child is declining overall.

**Fig 4:**
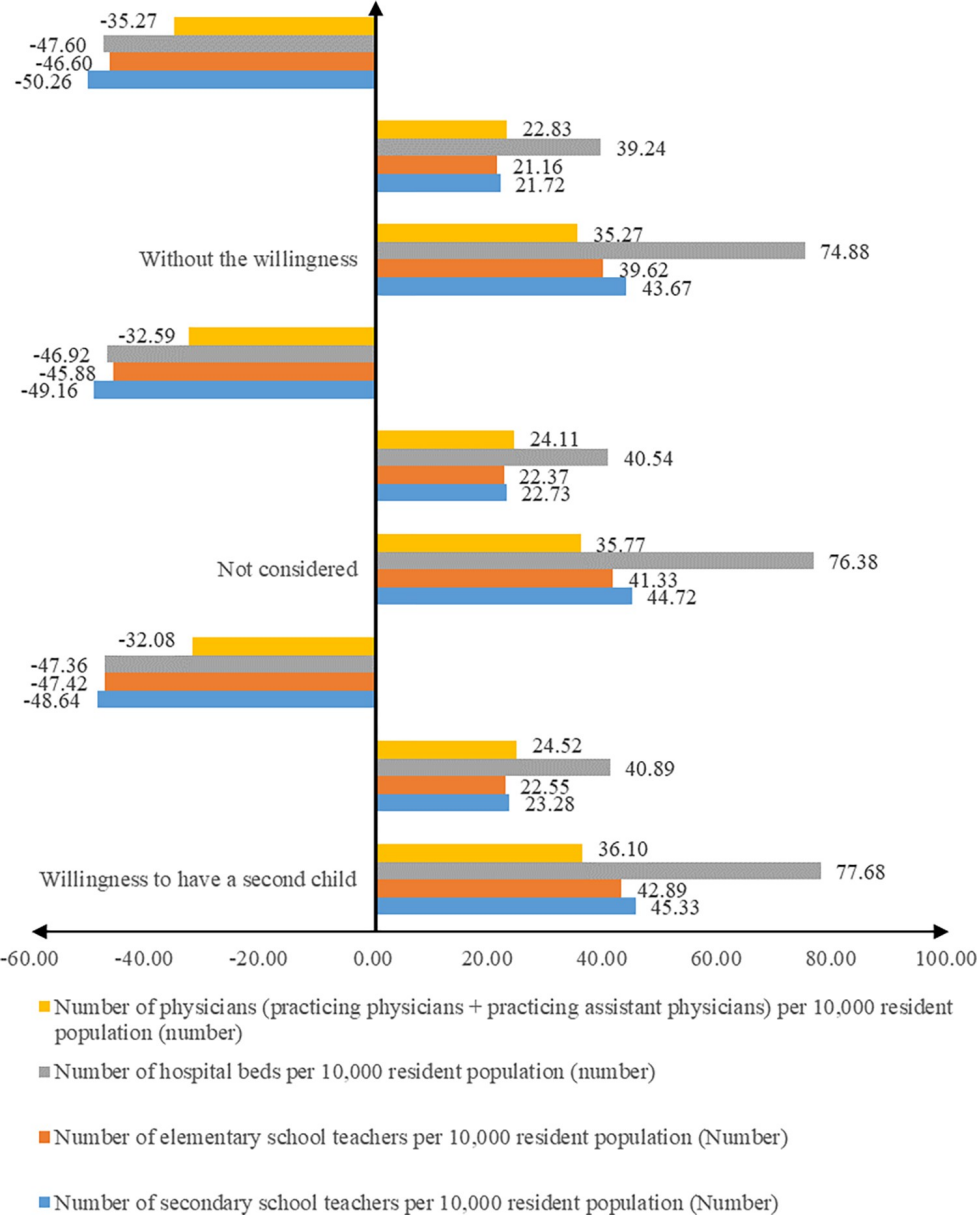
Comparison of public services owned by groups with different fertility intentions, 2016 and 2018. Note: Data from China Urban Statistical Yearbook, China Mobile Population Dynamic Monitoring Data, and China Provincial and Municipal Statistical Yearbook data. Calculation method of growth rate = (Average value of public services per 10,000 resident population in China in 2018—Average value of public services per 10,000 resident population in China in 2016) / Average value of public services per 10,000 resident population in China 2016 * 100. The table shows that (1), in general, the influx rate of the Migrant Population is much faster than the rate of the supply of urban public services, which has led to a continuous decline in the accessibility of urban public services for the Migrant Population. Accessibility of urban public services for the Migrant Population has been declining. (2) The accessibility of public services for the group that has the intention to have a second child > the accessibility of public services for the group that does not consider whether to have a second child > the accessibility of public services for the group that does not have the intention to have a second child.

Consequently, only samples comprising married individuals with one child within the childbearing age range (15–49 years old) were retained. 2016, there were 60,764 eligible survey data samples, with 43,167 eligible survey data samples in 2018. The specifics of the sample are shown in Figs 3 and 4.

### Measurement of variables

The independent variable considered in this study is the intention to have another child. Since fertility intentions do not entirely predict fertility behavior, there is often some degree of deviation between the two. This phenomenon is evident in many contemporary European and American countries, where the ideal family size is generally characterized by two children, yet fertility levels remain well below replacement levels. Similar discrepancies exist in Japan and Korea, which underwent demographic transitions earlier in Asia. In these countries, the reported ideal number of children in fertility surveys has remained stable at around two. However, the number of children born has declined, sometimes sharply, to nearly one child or even below.

Therefore, the explanatory variables in this paper include "whether you plan to have children in the next two years" and "whether you and your wife are currently using contraceptive methods," considering behavioral superposition. This is done to reflect the willingness of the Migrant Population to have children again. Social insurance, basic education, and primary medical care are selected as explanatory variables to measure the level of urban public services based on the first scores of principal component analysis, considering data accessibility and the results of principal component analysis. Additionally, this paper controls for urban characteristics, family characteristics, and personal characteristics to mitigate the potential impacts of these factors on the willingness of the Migrant Population to reproduce. The specific variables are calculated as shown in Table 2. See S1 Appendix for details of how the explanatory variables (Social security, Education, Medical Resources) were calculated.

**Table 2:** Calculation method of variables.

Categories	Variables	How to calculate variables
Explained variables	Willingness to have a second child	1. Willingness to not have a second child and use of contraceptive methods; 2. Willingness to not have a second child and no contraception; 3. Uncertain about a second child and use of contraceptive methods; 4. Uncertain about a second child and no contraception; 5. Willingness to have a second child and use of contraceptive methods; 6. Willingness to have a second child and use of contraceptive methods no contraception.
Explanatory variables	Social security	The first score of public services of urban security category
	fundamental education	First score value of principal component analysis of fundamental education
	Medical resources	First score value of principal component analysis of medical resources
Control variables- Factors of City Features	Level of economic development of the Urban	Take logarithm of GDP per capita for urban
	Urban salary level	Take the logarithm of the average annual wage for urban workers
	Urban industrial structure	Average value of tertiary sector/average value of secondary sector in urban areas
	Size of urban resident population	Take the logarithm of the resident population of the city
	City apartment prices	Take the logarithm of the average sales price per square meter of commercial housing in the urban area
	Level of city administration	1. Municipalities; 2. special economic zone; 3. sub-provincial city; 4. Provincial capital cities (Except for sub-provincial and special zones); 5. Other prefecture-level cities.
Control variables-Factors of family characteristics	Family members moving in with the situation	1. Family members (Spouse, children, parents) moving with; 0. Family members did not move with.
	Household rental expenses	1. Less than 500 yuan per month; 2. Above 500 yuan—below 1500 yuan per month; 3. More than 1500 yuan per month.
	Disposable income of the household	1. Less than 3000 yuan per month; 2. Above 3000 yuan—below 6000 yuan per month; 3. More than 6000 yuan per month.
	Gender of the first child	1. boy; 0. girl.
	Age of the first child	1. Age 0–3; 2. Age 4–6; 3. Age 7–10; 4. Age 11–14; 5. Age over 15.
	The first child’s current place of residence	1. The city that flows with family; 0. Other locations.
	Primary caregiver of the first child	1. Both parents; 2. Mother; 3. grandfather and grandmother; 4. Others.
Control variables-Factors of personal characteristics	Age	April 2016 (the time of the survey) minus the year of birth of the sample
	Age squared	Age* Age
	Gender	1. Male; 0. female.
	Level of education	0. Never been to school; 6. Finished elementary school; 9. Graduated from junior high school; 12. Graduated from high school or junior college; 14. Graduated from college; 16. Graduated from university with a bachelor’s degree; 19. Received a postgraduate bachelor’s degree; 22. Received a bachelor’s degree with a PhD.
	Nature of household registration	1. Agricultural household registration; 0. Non-agricultural household registration.
	Flowing area	1. Inter-provincial mobility; 0. Other types.
	Flowing time	1. Lessthan1year; 2. 1-2years; 3. 3-4years;4. 4-9years; 5. 10-14years; 6. 5–19 years; 7. 20–29 years; 8. Over 30 years.
	Willingness to settle down	1. Willingness; 2. Unwilling and unthinking.

Notes: This table reports the indicators for the specific measures of the explained, explanatory, and control variables and the way the variables were explicitly calculated. Non-agricultural household registration includes non-agricultural, agricultural to the resident, non-agricultural to the resident, and others. Other types contain inter-city and inter-county within the province.

## Empirical studies

### Basic regression models

This paper employs a fundamental multiple regression model, using the intention to have children again as the dependent variable and primary education and basic medical care as the independent variables. After accounting for individual, family, and city characteristics influencing the intention to have children again among the Migrant Population, the model is established.


$$Fertility=\beta_{0}+\beta_{1}P_{j}+\beta_{2}D_{j}+\beta_{3}x_{j}+\beta_{4}z_{j}+\varepsilon_{ij}$$ (1)

In the equation, the variable "Fertility" on the left-hand side is the dependent variable, while the variables on the right-hand side are the independent or explanatory variables. Furthermore, "Fertility" is a binary variable. When the migrant population in city j does not wish to have a second child, the value of "Fertility" is 0. When the migrant population in city *j* wishes to have a second child, the value of "Fertility" is 1. The core explanatory variable P indicates the level of basic security in city *j*, D indicates the level of development in city *j*, and ε is the disturbance term.

### Conditional Logit

The intention to have a second child exhibits variability across individual samples and programs. Consequently, this paper employs a Conditional Logit to analyze the heterogeneity within the migrant population, considering differences in human capital characteristics, employment features, and mobility traits. The constructed Conditional Logit model is presented below:

$$P(y_{i}=j|x_{ij})=\frac{\exp(x_{ij}^{'}\beta )}{\sum_{K}^{J}\exp(x_{ik}^{'}\beta )}$$ (2)

In the above equation, *p* indicates the probability that individual *i* chooses a different option for the intention to have a second child, and the explanatory variable *x*_*ij*_ indicates that it varies with sample individual *i* and also with option *j* (differences in human capital characteristics, differences in employment characteristics, and differences in mobility characteristics). The coefficient *β* indicates that the random utility of *p* does not depend on option *j*.

### Results

Table 3 presents the outcomes of stepwise regression analyses to have a second child as the dependent variable. Specifically, models (1), (2), and (3) showcase the results of stepwise regression estimation using 2016 data, while models (4), (5), and (6) illustrate the results based on the 2018 dataset.

**Table 3:** Comparative analysis of basic regression results.

Categories		Variables	Willingness to have a second child (2016)				Willingness to have a second child (2018)		
			(1)		(2)	(3)	(4)	(5)	(6)
Explanatory variables		Social security	0.001 (0.001)		0.001 (0.001)	-0.001 (-0.002)	0.000 (0.001)	0.000 (0.001)	0.001* (0.001)
		fundamental education	-0.154*** (-0.001)		-0.165*** (-0.011)	-0.140*** (-0.011)	0.004*** (0.001)	0.003*** (0.001)	-0.001*** (-0.011)
		Medical resources	-0.010*** (-0.001)		-0.012*** (-0.001)	-0.006*** (-0.002)	0.001*** (0.001)	0.003*** (0.001)	0.001*** (0.002)
Control variables-Factors of City Features		Level of economic development of the Urban				-0.047*** (-0.007)			-0.008*** (-0.001)
		Urban salary level				-0.014*** (-0.004)			-0.004*** (-0.001)
		Urban industrial structure				-0.014*** (-0.004)			-0.004*** (-0.001)
		Size of urban resident population				-0.013*** (-0.004)			-0.010*** (-0.001)
		City apartment prices				-0.099*** (-0.008)			-0.199*** (-0.007)
		Level of city administration				-0.012*** (-0.004)			-0.015*** (-0.003)
Control variables-Factors of family characteristics		Family members moving in with the situation			-0.025*** (-0.007)	-0.023*** (-0.001)		-0.005*** (-0.001)	-0.025*** (-0.000)
		Household rental expenses			-0.132*** (-0.005)	-0.152*** (-0.005)		-0.231*** (-0.005)	-0.253*** (-0.005)
		Disposable income of the household			-0.017*** (-0.003)	-0.005*** (-0.002)		0.127*** (0.004)	0.005*** (0.003)
		Gender of the first child			-0.101*** (-0.004)	-0.097*** (-0.004)		-0.301*** (-0.000)	-0.489*** (-0.000)
		Age of the first child			-0.001*** (-0.003)	-0.004*** (-0.003)		0.131*** (0.008)	-0.455*** (-0.001)
		The first child’s current place of residence			-0.01* (-0.005)	-0.003 (-0.006)		-0.000 (-0.005)	-0.009 (-0.002)
		Primary caregiver of the first child			-0.001* (-0.003)	-0.002* (-0.006)		-0.000 (-0.001)	-0.001* (-0.002)
Control variables- Factors of personal characteristics		Age	-0.005* (-0.003)		-0.012*** (-0.003)	-0.007** (-0.003)	-0.041*** (-0.002)	0.092*** (0.003)	-0.003** (-0.002)
		Age squared	-0.000*** (-0.000)		-0.000*** (-0.000)	-0.000*** (-0.000)	0.000*** (0.000)	0.000*** (0.000)	0.000*** (0.000)
		Gender	-0.007*** (-0.003)		-0.041*** (-0.000)	-0.029*** (-0.005)	-0.001*** (-0.003)	-0.003*** (-0.001)	-0.010*** (-0.003)
		Level of education	-0.002*** (-0.001)		-0.010*** (-0.001)	-0.001*** (-0.001)	0.011*** (0.001)	-0.100*** (-0.001)	-0.161*** (-0.001)
		Nature of household registration	-0.030*** (-0.005)		-0.031*** (-0.006)	-0.039*** (-0.006)	-0.009** (-0.003)	-0.110*** (-0.006)	-0.109*** (-0.006)
		Flowing area	-0.004*** (-0.004)		-0.001*** (-0.004)	-0.016*** (-0.005)	-0.013*** (-0.003)	-0.022*** (-0.003)	-0.028*** (-0.005)
		Flowing time	-0.005*** (-0.001)		-0.006*** (-0.002)	-0.005*** (-0.002)	-0.007*** (-0.001)	-0.009*** (-0.002)	-0.009*** (-0.002)
		Willingness to settle down	-0.013*** (-0.004)		-0.037*** (-0.004)	-0.037*** (-0.005)	0.005*** (0.005)	-0.007*** (-0.006)	-0.017*** (-0.005)
		Observations		60,764	45,371	35760	43167	39823	31678
		R^2^		0.084	0.07	0.11	0.092	0.081	0.12
		F		473.21	314.25	183.24	436.95	261.75	156.51

Notes

***, **, * indicate significance at the 1%, 5%, and 10% levels; data in brackets are robust standard errors. This study used non-standardized regression coefficients for estimation.

Through the comparison of regression results between 2016 and 2018, models (1) and (4) highlight the significant influence of personal characteristics on the willingness to have children among the Migrant Population. The regression outcomes indicate that, within the permissible age range for childbearing, the willingness to give birth to the next child increased significantly with the parent’s age in 2016; however, by 2018, older parents exhibited a declining inclination to have another child. This trend may be attributed to the release of short-term effects from the birth buildup and the higher medical and other childbearing costs for older parents. Men exhibit a significantly higher willingness to have more children than women. Higher-educated migrant populations also show a greater willingness to have children. Compared to non-agricultural household registration, migrant populations with agricultural household registration are more inclined to have another child. Those who do not move across provinces show a stronger intention to have another child compared to those moving across provinces. The duration of mobility is inversely proportional to the willingness to reproduce, while the willingness to settle down correlates positively to having another child.

Regarding urban public services, the regression coefficient indicates a low impact of essential security category services in the inflow city on the willingness of the mobile population to have another child. This aligns with earlier descriptive statistics illustrating the low access of the migrant population to public services in the inflow city, particularly in the realm of social insurance like pension and medical insurance. The preference for paying insurance in the place of domicile over the inflow area might be attributed to the migrant population’s prior contributions to New Rural Cooperative Insurance and New Rural Insurance in the inflow area. Women’s willingness to have more children decreases with age and is potentially linked to the physical condition of women at childbearing age.

The persistent preference for sons over daughters in China is evident, as families with boys exhibit a significantly lower willingness to have another child. This preference relates to the anticipated higher future costs of marriage and home purchase for boys in China. Notably, the caregiver’s gender for the first child plays a significant role, aligning with the traditional Chinese practice of females typically assuming the caregiving role. Families with a male caregiver for the first child express a lower intention to have another child. Economic factors influencing the willingness to have another child include employment status, with employees and self-employed individuals who often work overtime displaying a lower intention to have children. However, monthly household income, housing purchase, and pension insurance status do not show significant effects.

Lastly, house prices have a stable and significant effect on the willingness to have children among the Migrant Population. Models (3) and (6) indicate a negative correlation between housing prices and the propensity to have another child, suggesting that an increase in housing costs leads to a decrease in the willingness of mobile population to have another child. This conclusion is particularly pronounced in larger cities with higher house prices, emphasizing the substantial impact of renting costs on the fertility decisions of the migrant population.

## Heterogeneity analysis

### Characteristics of human capital

Does the impact of urban public services on the willingness of the migrant population to reproduce remain significant across various education levels and age groups? In addressing this, we categorized the migrant population according to their education levels and reproductive age in 2018. Education levels were classified into nine years and below and ten years and above based on the nine years of compulsory education. Age groups were divided into three categories based on ten years (as shown in Table 4).

**Table 4:** Regression results of the difference in human capital characteristics mobile population’s willingness to have another child.

Categories	Variables	Years of Education 9 years or less	Years of Education 10 years and above	Age group: 19–28	Age group: 29–38	Age group: 39–48
Explanatory variables	Social security	0.000** (0.000)	0.000** (0.000)	0.032** (0.013)	0.005 (0.009)	0.0711*** (0.023)
	fundamental education	0.987*** (0.1)	0.732*** (0.095)	0.795*** (0.125)	0.786*** (0.098)	1.113*** (0.223)
	Medical resources	0.0451** (0.018)	0.081*** (0.020)	0.003*** (0.001)	0.063*** (0.019)	0.129*** (0.043)
Control variables-Factors of City Features	Level of economic development of the Urban	0.300*** (0.060)	0.283*** (0.061)	0.166** (0.077)	0.378*** (0.06)	0.415*** (0.149)
	Urban salary level	-0.079** (-0.035)	-0.059 (-0.038)	-0.116*** (-0.044)	-0.064* (-0.038)	0.020 (-0.092)
	Urban industrial structure	0.086*** (-0.031)	0.114*** (-0.033)	0.152*** (-0.042)	0.084*** (-0.031)	0.114 (-0.075)
	Size of urban resident population	-0.080*** (-0.029)	-0.087*** (-0.032)	-0.080** (-0.038)	-0.049 (-0.031)	-0.184** (-0.076)
	City apartment prices	-0.581*** (-0.067)	-0.579*** (-0.062)	-0.575*** (-0.081)	-0.621*** (-0.064)	-0.396** (-0.17)
	Level of city administration	-0.074** (-0.035)	-0.130*** (-0.036)	-0.098** (-0.045)	-0.137*** (-0.035)	-0.053 (-0.092)
Control variables-Factors of family characteristics	Family members moving in with the situation	-0.159*** (-0.060)	-0.039 (-0.067)	-0.012 (-0.066)	-0.127* (-0.070)	-0.013 (-0.230)
	Household rental expenses	-0.216*** (-0.048)	-0.121*** (-0.042)	-0.164*** (-0.059)	-0.189*** (-0.043)	-0.001 (-0.111)
	Disposable income of the household	-0.117*** (-0.026)	-0.138*** (-0.027)	-0.121*** (-0.033)	-0.122*** (-0.027)	-0.068 (-0.070)
	Gender of the first child	-0.614*** (-0.038)	-0.526*** (-0.038)	-0.598*** (-0.048)	-0.554*** (-0.038)	-0.350*** (-0.101)
	Age of the first child	-0.094*** (-0.027)	-0.018 (-0.027)	-0.007 (-0.037)	-0.072*** (-0.024)	-0.571*** (-0.055)
	The first child’s current place of residence	-0.080 (-0.055)	-0.0427 (-0.048)	-0.068 (-0.067)	-0.027 (-0.051)	-0.036 (-0.125)
	Primary caregiver of the first child	-0.147** (-0.059)	-0.026 (-0.053)	-0.097 (-0.071)	-0.030 (-0.057)	-0.454*** (-0.139)
Control variables- Factors of personal characteristics	Gender	-0.223*** (-0.041)	-0.094** (-0.041)	-0.011 (-0.053)	-0.169*** (-0.040)	-0.418*** (-0.116)
	Nature of household registration	-0.317*** (-0.089)	-0.237*** (-0.046)	-0.029** (-0.012)	-0.018** (-0.009)	-0.083*** (-0.023)
	Flowing area	-0.140*** (-0.042)	-0.052 (-0.044)	-0.293*** (-0.088)	-0.297*** (-0.054)	-0.459*** (-0.137)
	Flowing time	0.025* (0.015)	0.051*** (0.016)	0.069*** (0.021)	0.026* (0.015)	-0.108*** (-0.032)
	Willingness to settle down	-0.269*** (-0.043)	-0.196*** (-0.041)	-0.164*** (-0.053)	-0.280*** (-0.041)	-0.248** (-0.109)
	Observations	20,130	15,630	8,256	15,196	8,643
	Pseudo R^2^	0.1604	0.0729	0.0359	0.0399	0.131
	Chi2	3325.4	1328.38	384.99	716.76	488.66

Notes

***, **, * indicate significance at the 1%, 5%, and 10% levels; data in brackets are robust standard errors. The Logitech model reports Pseudo R^2^ and Chi2 values. This study used non-standardized regression coefficients for estimation.

The educational landscape has significantly improved as China’s economy experiences ongoing development and societal progress. This shift fosters independent thinking, diminishing the inclination for childbearing and enhancing awareness of contraception, contributing to a reduced desire for additional children. Furthermore, childbearing introduces a heightened opportunity cost for women. Consequently, numerous scholars posit a strong correlation between the contemporary decline in fertility rates and the factors above. Conversely, there is a growing recognition among parents that investing in education represents a "long-term investment" characterized by steady growth. Drawing on Becker’s quantity-quality substitution model [68], parents are increasingly optimizing their choices, directing limited family resources and energies toward enhancing their children’s quality rather than quantity.

### Characteristics of the labor force

This paper categorizes the Migrant Population based on household income and occupational characteristics. Using the 25% and 75% quartiles, monthly household income is stratified into three groups: below RMB 4,000 for low-income, RMB 4,000 to RMB 8,000 for middle-income, and above RMB 8,000 for high-income. Occupational nature is differentiated into stable and non-stable employment. In alignment with the provided questionnaire options, stable employment includes individuals identified as heads of state organs, party and group organizations, enterprises and institutions, professional and technical personnel, civil servants, clerical staff, and related personnel. Others are classified as having non-stable employment(as shown in Table 5).

**Table 5:** Regression results in the willingness to have another child among the mobile population with differences in employment characteristics.

Categories	Variables	low income level	middle income level	high income level	stable employment	non-stable employment
Explanatory variables	Social security	-0.007 (-0.016)	-0.018** (-0.01)	-0.005 (-0.01)	-0.001 (-0.017)	-0.000* (-0.007)
	fundamental education	-0.971*** (-0.127)	-0.918*** (-0.109)	-0.707*** (-0.129)	-0.802*** (-0.236)	0.847*** (0.072)
	Medical resources	0.046* (0.025)	0.076*** (0.021)	0.062** 0.024)	0.0858* (0.045)	0.061*** (0.014)
Control variables-Factors of City Features	Level of economic development of the Urban	-0.357*** (-0.078)	-0.336*** (-0.067)	-0.188** (-0.082)	-0.187 (-0.129)	-0.307*** (-0.045)
	Urban salary level	-0.032 (-0.046)	-0.105** (-0.042)	-0.073 (-0.046)	-0.038 (-0.089)	-0.073*** (-0.027)
	Urban industrial structure	0.014 (0.041)	0.126*** (0.04)	-0.155*** (-0.044)	0.114* (0.068)	0.099*** (0.024)
	Size of urban resident population	0.132*** (0.04)	0.056 (0.035)	0.059 (0.039)	0.159** (0.073)	0.076*** (0.023)
	City apartment prices	0.712*** (0.103)	0.710*** (0.076)	0.449*** (0.071)	0.497*** (0.147)	0.596*** (0.048)
	Level of city administration	0.119** (0.052)	0.101** (0.034)	0.108** (0.043)	0.09 (0.082)	0.102*** (0.026)
Control variables-Factors of family Features	Family members moving in with the situation	0.078 (0.077)	0.151** (0.071)	0.066 (0.090)	0.418 (0.257)	0.086* (0.045)
	Household rental expenses	-0.183*** (-0.064)	-0.168*** (-0.05)	-0.163*** (-0.052)	-0.173* (-0.093)	-0.164*** (-0.033)
	Disposable income of the household	-0.488*** (-0.053)	-0.585*** (-0.043)	-0.623*** (-0.046)	0.130** (0.066)	0.122*** (0.02)
	Age of the first child	-0.065* (-0.037)	-0.041 (-0.031)	0.023 (0.034)	-0.553*** (-0.086)	-0.571*** (-0.028)
	The first child’s current place of residence	0.089 (0.070)	-0.05** (-0.138)	-0.106* (-0.062)	0.097 (0.062)	0.047** (0.020)
	Primary caregiver of the first child	0.047 (0.084)	0.138** (0.064)	0.077 (0.063)	0.005 (0.103)	-0.008 (-0.039)
Control variables- Factors of personal Features	Age	0.281*** (0.044)	0.489*** (0.042)	0.368*** (0.046)	0.041*** (0.113)	0.089* (0.042)
	Age squared	-0.006*** (-0.001)	-0.009*** (-0.001)	-0.007*** (-0.001)	-0.573*** (-0.102)	-0.376*** (-0.026)
	Gender	0.196*** (0.059)	0.168*** (0.046)	0.149*** (0.049)	0.010*** (0.002)	0.007*** (0.000)
	Level of education	-0.016 (-0.012)	-0.006 (-0.010)	0.016 (0.011)	0.075 (0.094)	0.187*** (0.031)
	Nature of household registration	0.219** (0.089)	0.277*** (0.067)	0.208*** (0.065)	0.148 (0.103)	0.267*** (0.046)
	Flowing area	-0.148** (-0.061)	-0.093* (-0.048)	-0.063 (-0.05)	-0.019 (-0.099)	-0.102*** (-0.032)
	Flowing time	-0.035* (-0.021)	-0.042** (-0.017)	-0.037** (-0.019)	-0.029 (-0.035)	-0.039*** (-0.011)
	Willingness to settle down	-0.263*** (-0.059)	-0.244*** (-0.046)	0.171*** (-0.05)	-0.283*** (-0.091)	-0.224*** (-0.031)
	Observations	9,762	15,005	10,993	3,178	32,582
	Pseudo R^2^	0.1254	0.128	0.1053	0.0761	0.1259
	Chi2	1283	2028.38	1359.39	273.1	4470.59

Notes

***, **, * indicate significance at the 1%, 5%, and 10% levels; data in brackets are robust standard errors. The Logitech model reports Pseudo R^2^ and Chi2 values. This study used non-standardized regression coefficients for estimation.

The regression findings reveal a noteworthy positive correlation between urban development public services and the inclination to have children among the migrant population, irrespective of their household income levels. However, the impact of urban public services on the willingness to have children is notably weaker in the high-income group due to their ability to choose between private and general products.

Whether the migrant population is engaged in stable or non-stable employment, primary education, and medical services significantly and positively influence their willingness to have children. In cases where the spouse works in a regular employment setting, such as a state-owned enterprise with fixed working hours, there is more time to assist with household chores and childcare. Increased spousal support positively correlates with a higher willingness of women to have another child. This effect is particularly pronounced among those in stable employment when a parent serves as the primary caregiver of the child (1 represents that the primary caregiver of the child is either the father or the mother, while 0 represents that the grandparents or other individuals serve as the primary caregiver.). Engagement in regular units, like state-owned enterprises, fosters a favorable environment for women’s fertility, reinforced by initiatives like maternity insurance and paid vacations. Given that stable employment migrants already benefit from social security provided by their workplaces (generally higher than that offered by flexible employment and urban workers types), the impact of urban universal social insurance on stable employment migrants is not statistically significant.

### Characteristics of the flow

Beyond individual endowments, encompassing human capital characteristics and employment stability, the reproductive intentions of the mobile population are also shaped by variations in mobility characteristics. In alignment with the standard procedure of the National Bureau of Statistics (NBS), geographic regions are classified into four groups: Eastern, Central, Western, and Northeastern regions. Additionally, the nature of household registration is stratified based on the place of outflow, distinguishing between urban-rural migrant populations and rural-rural migrant populations. These groupings serve as the basis for examining the influence of urban public services on the inclination of the migrant population to have another child, taking into account disparities in mobility(as shown in Table 6).

**Table 6:** Regression results in the willingness to have another child among the mobile population with differences in mobility characteristics.

Categories	Variables	Eastern Region	Middle Region	Western Region	Northeast Region	Mobility from urban to urban	Mobility from rural to urban areas
Explanatory variables	Social security	-0.011 (-0.009)	-0.056*** (-0.015)	-0.023* (-0.013)	-0.055 (-0.041)	-0.001 (-0.014)	-0.012* (-0.007)
	fundamental education	0.82*** (0.205)	0.013* (0.131)	0.605*** (0.137)	0.965 (3.838)	0.0919*** (0.174)	0.825*** (0.074)
	Medical resources	-0.02 (-0.033)	-0.017 (-0.021)	-0.068** (-0.028)	-0.873* (-0.446)	-0.080** (-0.034)	-0.057*** (-0.014)
Control variables-Factors of City Features	Level of economic development of the Urban	-0.312** (-0.132)	-0.194** (-0.089)	-0.395*** (-0.078)	-0.044 (-1.355)	-0.320*** (-0.106)	-0.252*** (-0.046)
	Urban salary level	-0.198*** (-0.069)	-0.014 (-0.071)	-0.001 (-0.05)	-0.282* (-0.678)	-0.0113 (-0.064)	-0.091*** (-0.028)
	Urban industrial structure	0.302*** (0.054)	0.062 (0.068)	0.141*** (0.036)	0.928 (1.273)	0.095* (0.054)	0.10*** (0.025)
	Size of urban resident population	-0.046 (-0.052)	-0.234*** (-0.053)	-0.070* (-0.037)	-0.74 (-0.804)	-0.156*** (-0.055)	-0.056** (-0.023)
	City apartment prices	-0.176*** (-0.068)	-0.834*** (-0.233)	-0.827*** (-0.183)	-1.759 (-0.374)	-0.633*** (-0.109)	-0.607*** (-0.049)
	Level of city administration	-0.004 (-0.043)	0.387** (0.18)	0.312*** (0.086)	0.703 (0.63)	0.133** (0.065)	-0.092*** (-0.027)
Control variables-Factors of family Features	Family members moving in with the situation	-0.188** (-0.079)	-0.045 (-0.086)	-0.144* (-0.076)	-0.085 (-0.26)	-0.088** (-0.078)	-0.085* (-0.046)
	Household rental expenses	-0.07 (-0.058)	-0.069 (-0.058)	-0.082 (-0.057)	-0.173 (-0.164)	-0.059 (-0.061)	-0.069 (-0.058)
	Gender of the first child	-0.718*** (-0.044)	-0.532*** (-0.053)	-0.522*** (-0.049)	-0.324** (-0.148)	-0.518*** (-0.044)	-0.534*** (-0.053)
	Age of the first child	-0.052* (-0.032)	-0.009 (-0.040)	-0.052*** (-0.035)	-0.089 (-0.107)	-0.056* (-0.032)	-0.009 (-0.040)
	The first child’s current place of residence	0.078 (0.059)	-0.051 (-0.071)	-0.034*** (-0.069)	-0.078 (-0.199)	0.068 (0.059)	-0.053 (-0.074)
	Primary caregiver of the first child	0.044 (0.062)	0.138* (0.08)	0.280*** (0.077)	0.178 (0.223)	0.056 (0.062)	0.135* (0.06)
Control variables- Factors of personal Features	Age	0.489*** (0.042)	0.435*** (0.051)	0.235*** (0.043)	0.323** (0.151)	0.318** (0.033)	0.056** (0.038)
	Gender	0.169*** (0.05)	0.149*** (0.057)	0.236*** (0.054)	0.394** (0.16)	0.465*** 0.05)	0.157*** (0.069)
	Level of education	-0.009 (-0.011)	-0.027** (-0.013)	-0.014 (-0.011)	-0.040 (-0.035)	-0.129** (-0.011)	-0.067** (-0.045)
	Flowing area	-0.266*** (-0.048)	-0.294*** (-0.066)	-0.076 (-0.057)	-0.383** (-0.159)	-0.281*** (-0.045)	-0.294*** (-0.066)
	Flowing time	-0.039** (-0.017)	-0.039* (-0.021)	-0.027 (-0.020)	-0.035 (-0.063)	-0.045** (-0.015)	-0.039* (-0.021)
	Willingness to settle down	-0.151*** (-0.046)	-0.143** (-0.061)	-0.320*** (-0.055)	-0.047 (-0.167)	-0.162*** (-0.046)	-0.143** (-0.061)
	Observations	12,209	9,507	10,198	3,846	12,209	9,507
	Pseudo R^2^	0.1262	0.1025	0.1146	0.119	0.1987	0.1348
	Chi2	1867.06	1035.18	1308.81	191.9	1968.34	989.34

Notes

***, **, * indicate significance at the 1%, 5%, and 10% levels; data in brackets are robust standard errors. The Logitech model reports Pseudo R^2^ and Chi2 values. This study used non-standardized regression coefficients for estimation.

Irrespective of the geographical area to which the mobile population has migrated, the primary education index exhibits a significant positive correlation with their willingness to have another child. Notably, the pronounced impact of the primary education index is observed in the eastern region, with a continuous marginal effect of 0.82 at the mean of the primary education index. This implies that for every 1% increase in the primary education index, the probability of the mobile population’s desire to have another child increases by 0.82 percentage points. The influence of urban public services is most prominent in the West but relatively weaker in the Northeast. This discrepancy might be attributed to the Northeast experiencing lesser effects from social security factors due to its higher education level, substantial outflow of individuals of childbearing age, and fewer ethnic minorities.

Additionally, it could be linked to regional marital cultures and economic conditions. Among the influencing factors, primary education has the most substantial impact on the migrant population in the eastern region, possibly stemming from a population concentration that results in a relative scarcity of essential education resources. Meanwhile, the medical service factor exerts the most decisive influence on the mobile population in the western region, likely associated with the lagging medical infrastructure in the area, accentuating the significance of medical service resources.

For both urban-city and rural-city mobile populations, urban development-type public services exhibit a significant positive relationship with their willingness to have another child. However, it is evident that urban public services more strongly influence the reproductive intentions of the urban-city mobile population. Scholars addressing the lack of social security for urban-city and rural-urban migrant populations highlight the double disadvantage faced by the rural-urban migrant population, creating significant disparities in status and opportunities with the urban-rural migrant population. The latter group, being selectively mobile, benefits from higher education levels and skills, which are advantageous for their entry into the inflow area, offering advantages in employment choices, career progression, income levels, and networking resources, thereby enhancing their social security. In contrast, the rural-urban migrant population encounters inherent disadvantages, such as lower education levels and a lack of skills, which prevent them from enjoying the benefits of local citizenship and participating in the household registration-based social security system. Simultaneously, their acquired deficiencies place them at a disadvantage in choosing professions, further widening the gap between them and developmental aspects of public services like health insurance and primary education.

## Robustness test

To enhance the reliability of the aforementioned findings, this study utilizes two distinct methodologies for testing. Firstly, the urban public services indicator in the 2016 data is replaced. Secondly, the 2018 data is subjected to a test where the sample of individuals "not considering whether to have two children" is excluded. Both robustness tests consistently affirm the model’s strong fit. The results indicate that, even with the transformation of relevant indicators, the inclination of the Migrant Population to have a second child is significantly influenced by the essential public services in the city. Notably, while these results remain broadly consistent with the overall model, there are variations in the extent of the impact.

(1) Robustness test for sample error in explanatory variables. Throughout this paper, samples indicating no consideration for another child are treated as lacking the intention to have one during the research process. However, it is imperative to acknowledge that not contemplating it does not equate to a complete absence of intention; individuals may still possess the desire to have another child. To enhance rigor, categorical variables are reset by excluding samples falling under categories 3 (those not wanting children and using contraception) and 4 (those not wanting children and not using contraception), resulting in 11,731 samples. The estimation and analysis are then conducted exclusively on samples explicitly expressing their intention to have another child. The table below illustrates that, regardless of the inclusion or exclusion of characteristic variables from other cities, the two-child reproduction intention of the migrant population is significantly impacted by urban public services. This reaffirms the robustness of the findings, indicating that higher levels of urban public services strengthen the willingness of the migrant population to have another child(as shown in Table 7).

**Table 7:** Regression results after excluding some samples.

Variables	logit(1)	logit(2)
Per capita public finance expenditure	-0.013(-0.018)	0.272***(0.039)
Number of teachers per 10,000 elementary school students	0.246***(0.086)	0.162***(0.096)
Number of doctors per 10,000 resident population	0.202***(0.027)	0.391***(0.041)
Level of economic development of the Urban		-0.616***(-0.053)
Urban salary level		-0.185***(-0.031)
Urban industrial structure		-0.234***(-0.028)
Size of urban resident population		-0.0455(-0.028)
City apartment prices		-0.564***(-0.061)
Level of city administration		-0.101***(-0.03)
Age	-0.331***(-0.024)	-0.341***(-0.029)
Age squared	0.008***(0.000)	0.008***(0.000)
Gender	0.434***(0.028)	0.388***(0.034)
Level of education	0.036***(0.006)	0.046***(0.007)
Nature of household registration	0.173***(0.039)	0.274***(0.048)
Flowing area	-0.060**(-0.028)	-0.134***(-0.035)
Flowing time	-0.012(-0.01)	-0.013(-0.012)
Willingness to settle down	0.121***(0.028)	0.156***(0.034)
Disposable income of the household	0.055***(0.019)	0.116***(0.022)
Family members moving in with the situation	0.196***(0.044)	0.129**(0.052)
Household rental expenses	-0.178***(-0.03)	-0.185***(-0.036)
Observations	35,881	24,952
PseudoR^2^	0.2178	0.2425
Chi2	9950.91	7813.4

Notes

*, **, *** indicate significant at the 10%, 5%, and 1% levels, respectively; data in parentheses are robust standard errors; Logitech models report PseudoR^2^ and Chi2 values. This study used non-standardized regression coefficients for estimation.

(2) Robustness test for computational errors of core explanatory variables. In this study, urban public services in the inflow area are generally classified into three evaluation modes per the related subject literature. These include the objective input level of public services, primarily comprising local public service expenditures or infrastructure construction expenditures; the objective output level of public services, encompassing qualitative changes in the provision of public products by primary and secondary schools, public hospitals, etc. as well as indicators measuring the quality of public services, such as the academic qualifications of full-time teachers, and the individual’s subjective evaluation of the city’s public services, which involves questionnaire surveys, among other methods. This paper synthesizes the first and second evaluation models, focusing on urban public services’ objective input and output models. A single indicator is employed to replace the composite indicator, testing the robustness of the basic econometric model. Per capita fiscal expenditure substitutes for basic security public services, while the number of teachers per 10,000 elementary school students and doctors per 10,000 resident population replaces primary education and medical services. The robustness test results are presented in the table: irrespective of whether other city characteristic variables are included in the regression results, urban public services consistently exhibit a significant positive effect on the two-child re-birth intention of the migrant population. This aligns with the fundamental regression results of this paper and further validates the robustness of the findings (as shown in Table 8).

**Table 8:** Robustness tests for replacing public service indicators.

Variables	logit(1)	logit(2)
Per capita public finance expenditure	-0.004(-0.012)	0.168***(0.033)
Number of teachers per 10,000 elementary school students	0.860***(0.073)	0.809***(0.081)
Number of doctors per 10,000 resident population	0.150***(0.023)	0.273***(0.034)
Level of economic development of the Urban		-0.387***(-0.043)
Urban salary level		-0.097***(-0.025)
Urban industrial structure		0.187***(0.023)
Size of urban resident population		0.0453*(0.023)
City apartment prices		0.378***(0.049)
Level of city administration		0.121***(0.025)
Age	0.363***(0.021)	0.372***(0.024)
Age squared	-0.007***(-0.000)	-0.007***(-0.000)
Gender	0.250***(0.023)	0.203***(0.028)
Level of education	0.006(0.005)	0.010*(0.006)
Nature of household registration	0.147***(0.033)	0.210***(0.04)
Flowing area	-0.040*(-0.024)	-0.106***(-0.029)
Flowing time	0.031***(0.009)	0.032***(0.01)
Willingness to settle down	0.194***(0.024)	0.222***(0.029)
Disposable income of the household	0.067***(0.012)	0.107***(0.019)
Family members moving in with the situation	0.170***(0.037)	0.111***(0.043)
Household rental expenses	-0.144***(-0.026)	-0.140***(-0.031)
Observations	52,545	36,742
PseudoR^2^	0.0975	0.1073
Chi2	5494.8	4303.89

Notes

*, **, *** indicates significant at the 10%, 5%, and 1% levels, respectively; data in parentheses are robust standard errors; Logitech models report PseudoR^2^ and Chi2 values. This study used non-standardized regression coefficients for estimation.

## Conclusion and discussion

In conclusion, this study offers valuable insights into the impact of urban public services on the willingness of the migrant population to have more children. These findings have sparked significant discussions within the realms of urbanization, fertility behavior, and public service provision, enriching academic discourse and further exploring the implications of these results.

Firstly, the positive correlation observed between essential public services in the domain of urban security and the increased willingness of the Migrant Population to reproduce aligns with the views of scholars who emphasize the role of social security as a driver of population dynamics [69]. As highlighted by Zhou [43], the evolving landscape of social security often plays a critical role in shaping population decisions, acting as a substitute for traditional family support structures [70].

Secondly, the identified positive correlation between primary education and the willingness to have children is in accordance with assertions made by scholars [71] regarding the influence of educational investments on altering fertility preferences. This underscores the socio-economic dimensions of fertility decision-making, where improved educational opportunities can mitigate perceived costs and enhance the desirability of reproduction.

Thirdly, the observed positive correlation between essential medical services and the willingness to have children is consistent with the framework proposed by Ji [72], emphasizing the crucial role of health-related services in shaping fertility intentions. Comprehensive and accessible medical services can address reproductive health issues and create an environment conducive to family expansion.

These findings raise intriguing questions about the evolving role of urban public services in a dynamic and ever-changing society. The counterintuitive impact of urban public services on the willingness of the Migrant Population to reproduce challenges conventional perspectives regarding the positive role of public services in demographic dynamics. This phenomenon can be interpreted through the lens of Polanyi theory [73], which suggests that urbanization might induce shifts in individual values and lifestyle choices, thus influencing fertility decisions.

The differential impact of security, medical services, and primary education on increasing fertility intentions deserves further exploration. As proposed by scholars [74], these varying effects may be attributed to differences in the perceived importance of these services in the lives of the Migrant Population [75]. Future research should delve deeper into the qualitative aspects of these services to unveil potential underlying mechanisms.

In summary, the theoretical significance of these findings extends beyond the scope of this study, opening up new avenues for research to delve into the intricate relationship between urban public services and fertility preferences within the context of contemporary urbanization dynamics.

## Supporting information

S1 Appendix Construction of indicators for urban basic public services. (DOCX)
