# Supplementary material for: Urban public services and fertility intentions of internal migrants in China
Source: PLoS One. 2024 Mar 28;19(3):e0300345. doi: 10.1371/journal.pone.0300345 (PMC10977766; doi:10.1371/journal.pone.0300345)
Supplement: S1 Appendix — (DOCX) [file pone.0300345.s001.docx]

**Appendix 1:**

**Construction of indicators for urban basic public services**

According to the "narrow school" perspective on basic public services advocated by scholars, this paper proposes the inclusion of public education, public health care, and social security—services reflecting citizens' rights—within the equalization category. The classification of urban basic public services is further delineated into the realms of protection and development.

In this study, the security category's core variable gauges the floating population's access to public services through three dimensions: social insurance, employment security, and housing security. Firstly, the social insurance variable considers the migrant population's limited human capital and scarce social resources in the inflow city compared to urban residents. This potential scarcity may lead to challenges such as income loss due to illness, maternity, unemployment, and work-related injuries, impacting their ability to sustain themselves. The study explores the impact of providing five basic social guarantees—old-age pension, medical care, unemployment benefits, work-related injury compensation, and maternity benefits—in the inflow city on the willingness of the migrant population to have children.

Secondly, the employment security variable addresses the vulnerability of the migrant population in the labor market due to job instability, limited qualifications, and training opportunities. The lack of legal constraints between migrant workers and employers often results in disputes and losses for the former. The study measures the impact on the willingness to have another child based on the presence of a signed labor contract and the establishment of a health record.

Thirdly, the housing security variable underscores the pivotal role of housing in facilitating the access of migrant children to basic education resources. The study examines whether the employer provides accommodation, the number of individuals accommodated, and the discounted market cost of accommodation. Given the challenging economic affordability of the migrant population amid soaring urban housing prices, this variable becomes crucial in understanding their willingness to have children.

To address covariance among variables and maximize relevant information retention, this paper employs principal component analysis to comprehensively calculate the safeguarded public service index, serving as a measure of the urban public services level. The weights assigned to the indicators (as detailed in Table 1) emphasize the significance of basic social insurance, aligning with the core service provided by the city. The total weights for social insurance and employment security are 12%, while housing security holds a 15% weight within the guaranteed public services, reflecting their relative importance.

**Table 1: Composition and weights of the guaranteed public service indicator system**

| Level 1 Indicators | Level 2 Indicators | Weights |
| --- | --- | --- |
| Social security | Whether to participate in the inflow of pension insurance | 0.12 |
|  | Whether to participate in the inflow of medical insurance | 0.13 |
|  | Whether to participate in the inflow of work injury insurance | 0.16 |
|  | Whether to participate in the inflow of unemployment insurance | 0.16 |
|  | Whether to participate in the inflow of maternity insurance | 0.16 |
| Security of employability | Whether to sign an employment contract | 0.11 |
|  | Whether to establish a health record | 0.01 |
| Security of housing | Whether there is an employer to provide accommodation | 0.13 |
|  | Whether there is an employer to provide accommodation for one or more people | 0.02 |

Notes：Data from the China City Statistical Yearbook. The first component scores with eigenvalues greater than 1 in this table have a principal component variance contribution of 67.26%. Because of the negative values of principal components, the first score value plus 3 was adjusted for the convenience of the study.

In the preceding literature, the research focus within the urban development category of public services is delineated in Table 2. Notably, the weights assigned to basic education and medical services are 47.79% and 21.03%, respectively, aligning with societal expectations of urban development public services. Furthermore, the interconnectedness of basic education and medical services with the willingness of the migrant population to have children again underscores their relevance. To ensure precision in measuring public services (Qiao Baoyun et al., 2005), this study employs various indicators. Specifically, the level of basic education is comprehensively assessed using metrics such as the number of full-time teachers per 10,000 secondary school students, the number of full-time teachers per 10,000 elementary school students, the number of schools per 10,000 secondary school students, and the number of schools per 10,000 elementary school students. Concurrently, the evaluation of medical services encompasses indicators like the number of hospitals per 10,000 residents, the number of beds per 10,000 residents, and the number of doctors per 10,000 residents. To mitigate covariance issues arising from the correlation between basic education and medical service variables, the paper adopts the principal component analysis method. This involves deriving the first principal component score values for basic education and medical service, with an additional adjustment of 3 to render them positive for the sake of consistency with the explanatory variables in the protection category.

**Table 2: Composition and weights of the public service indicator system for development category**

| Level 1 Indicators | Level 2 Indicators | Weights |
| --- | --- | --- |
| fundamental education | Number of teachers per 10,000 secondary school students (persons) | 0.4779 |
|  | Number of teachers per 10,000 elementary school students (persons) |  |
|  | Number of schools per 10,000 secondary school students (schools) |  |
|  | Number of schools per 10,000 elementary school students (schools) |  |
| medical resources | Number of hospitals per 10,000 people (number) | 0.2103 |
|  | Number of hospital beds per 10,000 people (beds) |  |
|  | Number of doctors per 10,000 people (persons) |  |
| Urban Transportation | Road area per capita (square meters) | 0.1491 |
|  | Number of buses per 10,000 people (units) |  |
|  | Number of rental cars per 10,000 people (units) |  |
| Urban Environment | Greening coverage of built-up areas (%) | 0.1329 |
|  | Comprehensive utilization rate of industrial solid waste (%) |  |
|  | Centralized sewage treatment rate (%) |  |
|  | Harmless treatment rate of domestic waste (%) |  |
| Urban Culture | Number of books in public libraries per 10,000 people (volumes, pieces) | 0.03 |
|  | Culture, sports and entertainment employees per 10,000 people (persons) |  |

Notes:Data from the China City Statistical Yearbook.

According to the above construction of urban basic public service indicators, this paper selects social insurance, fundamental education, and medical resources as important indicators to measure the level of urban public services.
